# Supplementary material for: The interactome of intact mitochondria by cross-linking mass spectrometry provides evidence for coexisting respiratory supercomplexes
Source: Mol Cell Proteomics. 2017 Dec 8;17(2):216–32. doi: 10.1074/mcp.RA117.000470 (PMC5795388; doi:10.1074/mcp.RA117.000470)
Supplement: Supplemental Data [file supp_RA117.000470_133922_0_supp_23978_fzffwf.zip › spectra_annotation/mito_DR_spectra_annotation/308-1-10-1-5-1.pdf]

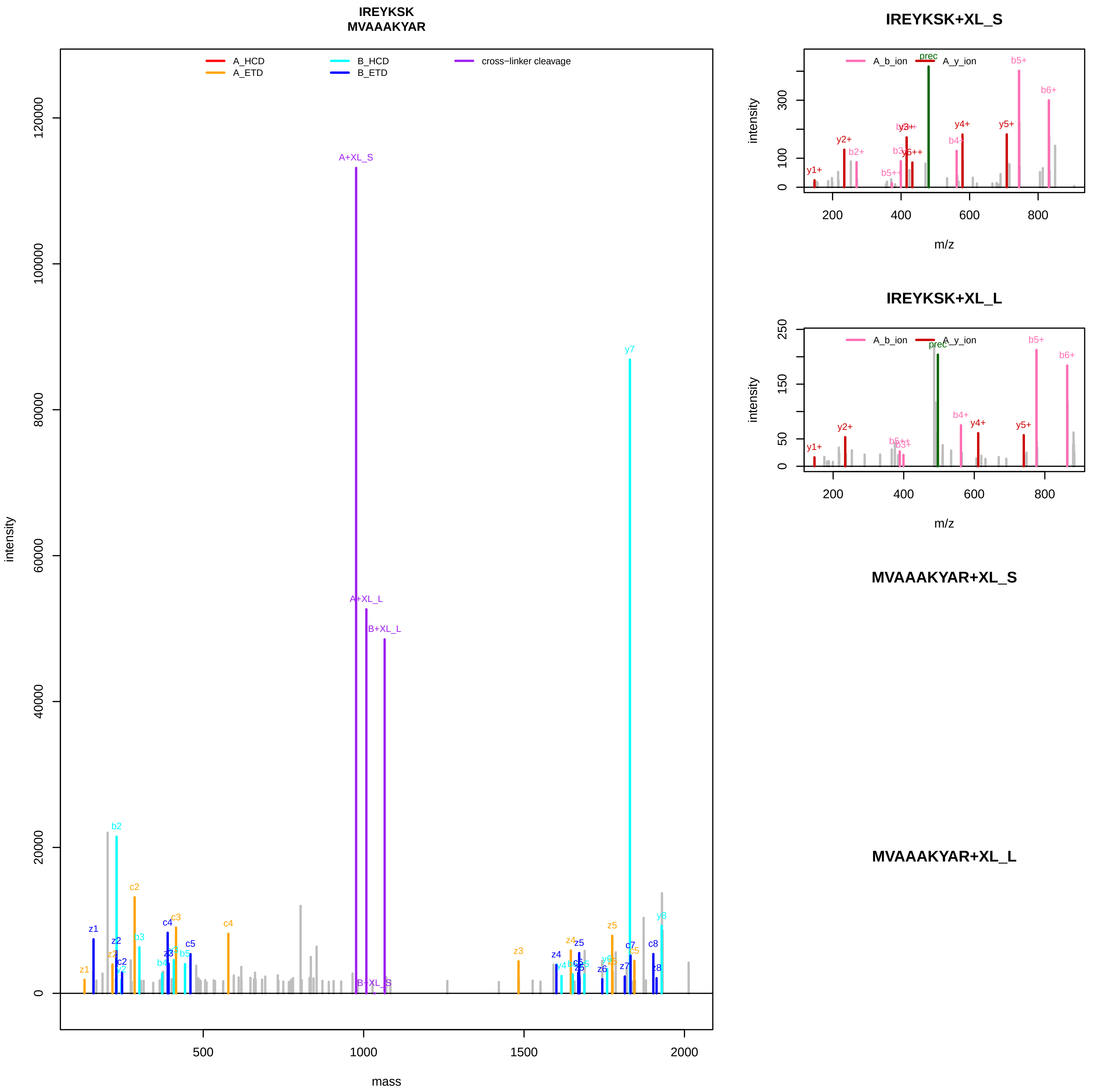
IREYKSK
MVAAAKYAR
A_HCD
A_ETD
B_HCD
B_ETD
cross−linker cleavage
A+XL_S
A+XL_L
B+XL_L
B+XL_S
y7
y8
b2
b3
b4
b5
c2
c3
c4
c5
c7
c8
z1
z2
z3
z4
z5
z6
z7
z8
intensity
mass
0
20000
40000
60000
80000
100000
120000
500
1000
1500
2000
IREYKSK+XL_S
IREYKSK+XL_L
A_b_ion
A_y_ion
prec
y1+
y2+
y3+
y4+
y5+
y6++
b2+
b3+
b4+
b5+
b6+
b5++
m/z
MVAAAKYAR+XL_S
MVAAAKYAR+XL_L
